# Concurrent somatic mutations in driver genes were significantly correlated with lymph node metastasis and pathological types in solid tumors

**DOI:** 10.18632/oncotarget.19975

**Published:** 2017-08-07

**Authors:** Yanan Cheng, Shaojing Wang, Lei Han, Pengpeng Liu, Hui Li, Xiubao Ren, Jinpu Yu, Xishan Hao

**Affiliations:** ^1^ Cancer Molecular Diagnostics Core, Tianjin Medical University Cancer Institute and Hospital, National Clinical Research Center for Cancer, Key Laboratory of Cancer Prevention and Therapy, Tianjin 300060, China; ^2^ Tianjin's Clinical Research Center for Cancer, Tianjin 300060, China; ^3^ Tianjin Novcare Biotech., Ltd., Tianjin 300300, China; ^4^ Department of Gastrointestinal Cancer Biology, Tianjin Medical University Cancer Institute and Hospital, National Clinical Research Center for Cancer, Key Laboratory of Cancer Prevention and Therapy, Tianjin 300060, China; ^5^ Tianjin's Clinical Research Center for Cancer, Tianjin 300060, China; ^6^ Biotherapy Center, Tianjin Medical University Cancer Institute and Hospital, National Clinical Research Center for Cancer, Key Laboratory of Cancer Prevention and Therapy, Tianjin 300060, China; ^7^ Tianjin's Clinical Research Center for Cancer, Tianjin 300060, China

**Keywords:** NGS, tumor genetic profiling, concurrent somatic mutations, solid tumors, NSCLC

## Abstract

To demonstrate the mutational profiles in solid tumors, we profiled 165 solid tumor samples, including 9 cancer types and 4 sample types, by using amplicon-based next-generation sequencing panel covering 48 highly mutated tumorigenesis-related genes that were deep sequenced at an average coverage of 2000×. Both tumor and sample types had significant effect on tumor genetic mutational profiles. Concurrent driver mutations were frequently detected in solid tumor, concentrating on both modes of action driver genes (activating or loss of function). Furthermore, in non-small cell lung cancer (NSCLC), concurrent driver mutations were also significantly correlated with the lymph node metastasis status and pathological types. Higher frequency of lymph node metastasis was observed in patients with NSCLC with concurrent mutations on at least two driver genes. In addition, patients with lung adenocarcinoma were more likely to harbor concurrent driver mutations than patients with lung squamous and large cell carcinoma. Multiple mutations in the epidermal growth factor receptor gene were more frequently detected in patients with refractory NSCLC compared to untreated naive ones. Therefore, concurrent multiple driver mutations, rather than a single genetic mutation, should be investigated extensively to probe novel genetic biomarkers with clinical benefits.

## INTRODUCTION

Cancer treatment has made paradigm shift advancements in the past decade with the development of therapies targeting specific genetic alterations. A number of target therapies have shown great antitumor efficiency in the clinic, such as the epidermal growth factor receptor–tyrosine kinase inhibitors (EGFR-TKI) gefitinib and erlotinib for patients with non-small cell lung cancer (NSCLC) harboring sensitizing EGFR mutations [1], crizotinib for patients with NSCLC bearing ALK and ROS1 fusion [1, 2], and Gleevec for patients with gastrointestinal stromal tumor with mutated KIT [3]. Tumor genotyping makes it possible to categorize patients into different subgroups and to treat them with the optimal regimens to achieve more satisfactory therapeutic effects. Therefore, in the newly released National Comprehensive Cancer Network Guidelines of 2017, broad genetic diagnostic tests have been recommended for primary patients with NSCLC to determine the best first-line therapeutic regimen [4].

DNA next-generation sequencing (NGS) is a large-scale parallel sequencing technology and has been proven as an accurate tool to detect various forms of genetic abnormalities, including mutations, fusions, and amplifications, across a large number of genes in a high-speed and high-efficiency pattern [5].

Compared with whole genome NGS, targeted NGS focuses on the specific regions containing cancer-relevant genes, has lower cost, and generates more reliable and usable data due to high coverage of targeted regions. Tumor always shows strong heterogeneity, some low-frequency driver mutations can exclusively be detected by deep sequencing because patients carrying these low-frequency mutations are also sensitive to targeted therapy. Targeted NGS is efficient and accurate in screening drug targets and provides more treatment options for patients with cancer, especially patients with advanced stage cancer who cannot tolerate the side effects of chemotherapy.

Development of targeted NGS technology resulted in more attention to be given in identifying cancer-related driver gene pathways, which precisely illustrate how different driver genes collaborate simultaneously to induce carcinogenesis and progression [6, 7]. Specific combinatorial patterns of genetic mutations are significantly correlated with certain tissue type and patient prognosis [8]. However, combinatorial patterns in most solid tumors are not yet identified. Therefore, in this study, we focused on the mutational profiling of 48 highly mutated genes in 165 solid tumor tissue samples across 9 common solid tumor types using TruSeq Amplicon Cancer Panel (TSACP). We also investigated the distribution of concurrent mutations among different genes to define accurate cancer molecular subtypes, which will help oncologists to prescribe optimal therapeutic regimens and precisely predict clinical outcome after the treatment.

We found that tumor and sample types had significant effect on tumor genetic mutation profiling. Concurrent somatic driver mutations were frequently detected in solid tumor tissues, and most of the concurrent mutations concentrated on both modes of action driver genes (activating and loss of function), which significantly correlated with the lymph node metastasis status, pathological types, and clinical response to therapies in NSCLC. Therefore, NGS is a reliable method to detect concurrent multiple driver mutations, which rather than a single genetic mutation, should be investigated more extensively to probe novel genetic biomarkers with clinical predictive benefits.

## RESULTS

### TSACP assay is a reliable method to detect multiplex mutations in tumors

**Figure 1:**
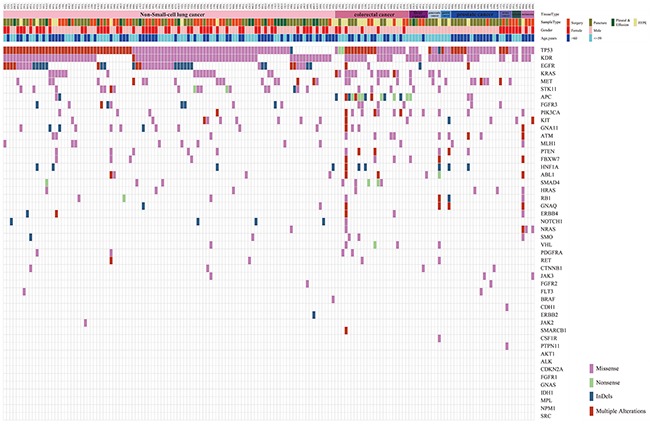
Distribution of 766 passed filtering variants The top of the figure shows the tissue type, sample type, gender, and age for 165 samples. The body of the figure shows 766 variants distributed based on the sample ID and gene name.

Profiling of 165 solid tumor tissue samples was performed via amplicon-based target sequencing using Illumina TSACP assay. Basic quality control of raw data was conducted, and the following findings were observed: the average sequencing depth reached 2000×; all samples had Q30 percentage higher than 80%; the average ratio of reads mapped to target regions was 96%; and the average percentage of sequencing uniformity (the proportions of sequences that have greater than 0.2 times the mean coverage [9]) reached 91%. The sequencing data were qualified and sufficient for further bioinformatics analysis. A total of 8526 variants were identified in 165 solid tumor samples, among which 6174 variants were located in noncoding regions and 1194 variants were synonymous variants. Afterward, 1158 missense/nonsense SNVs and InDels were kept. Considering the lowest detectable threshold of TSACP assay, another 392 variants with allele frequency lower than 3% were discarded for uncertain positive evidence. Finally, 766 variants, which were important for the following analysis, were determined. Figure 1 shows the distribution of 766 variants. The number of genetic variants detected in each sample varied among different tumor and sample types, which ranged from 1 to 21 variants per sample with an average of 5.6 variants per sample. Furthermore, various types of mutations, including both SNVs and InDels, occurred in 39 genes covering 81.25% (39/48) of the known highly mutated genes in cancer. The most frequently mutated genes were TP53 (88.48%), KDR (72.73%), EGFR (30.1%), KRAS (20.0%), and MET (18.97%).

### Mutations were distributed variously among different tumor types

**Figure 2:**
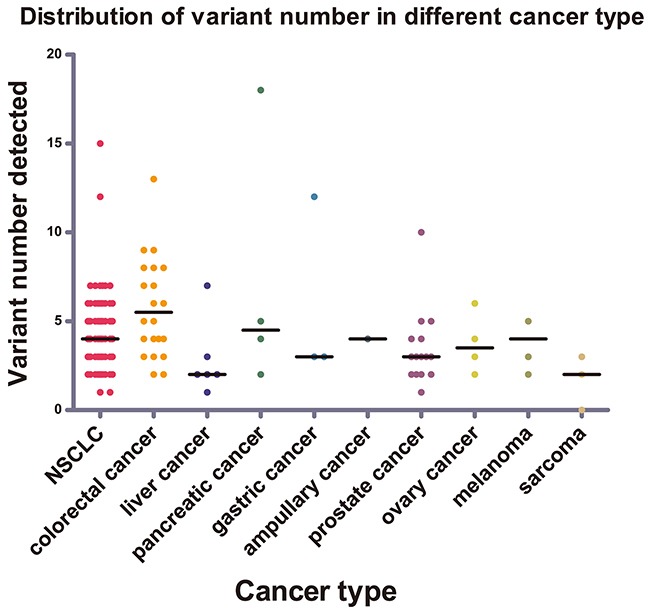
Distribution of variant numbers in different cancer types The variant numbers of each sample from nine different cancer types were marked with different colors. The black lines were the median number for variant number of the corresponding cancer types.

Nine kinds of tumor types were included in this study. Different tumor types displayed distinct genetic mutation profilings (Figure 2). In NSCLC, the most frequently mutated genes were TP53 (87.38%), KDR (78.64%), EGFR (44.66%), STK11 (17.48%), MET (16.5%), and KRAS (15.53%). In colorectal cancer (CRC), the most frequently mutated genes were TP53 (95.65%), APC (60.87%), KDR (56.52%), KRAS (56.52%), and PIK3CA (30.43%). In prostate cancer (PsC), the most frequently mutated genes were TP53 (93.33%), KDR (53.33%), MET (33.33%), HRAS (13.33%), APC (13.33%), and ATM (13.33%) (Supplementary Table 1). Our result in NSCLC was in accordance with the published Asian data, such as the EGFR mutation frequency around 50% [10]. Additionally, our CRC mutation frequencywas slightly higher than the reported TCGA data in common [11].

The mutation loads varied among different tumor types. We compared the amount of mutations among the three major tumor types, including NSCLC, CRC, and PsC. Statistically, the amount of mutations per sample of CRC (median, 5; range, 2–13) was comparably higher than that of PsC (median, 3; range, 1–10) with a p value of 0.004 by nonparametric test. Although the amount of mutations per NSCLC sample (median, 4; range, 1–15) was lower than that of CRC sample, no statistically significant difference was determined. In addition, among the different tumor types of the digestive system, the amount of mutations per CRC sample was relatively higher than the others (median, 3; range, 1–12), including hepatic cancer (HC), pancreatic cancer (PcC), and gastric cancer (GC), with a p value of 0.022 by nonparametric test. We determined that fewer mutations exist in mesenchymal tissue-derived tumors than in epithelial-derived cancers, in which the amount of mutations per sarcoma sample was less than 3; this result hence suggested that the histological origin had a significant effect on tumor mutation profiling based on TSACP assay. Besides, TSACP is a small-scale panel, the mutation frequency of 48 detected genes may underestimate the mutation abundance of PsC and mesenchymal/sarcoma mutations, which show the unique profile of cancer mutations [12–15].

### Mutations were distributed variously among different sample types

**Figure 3:**
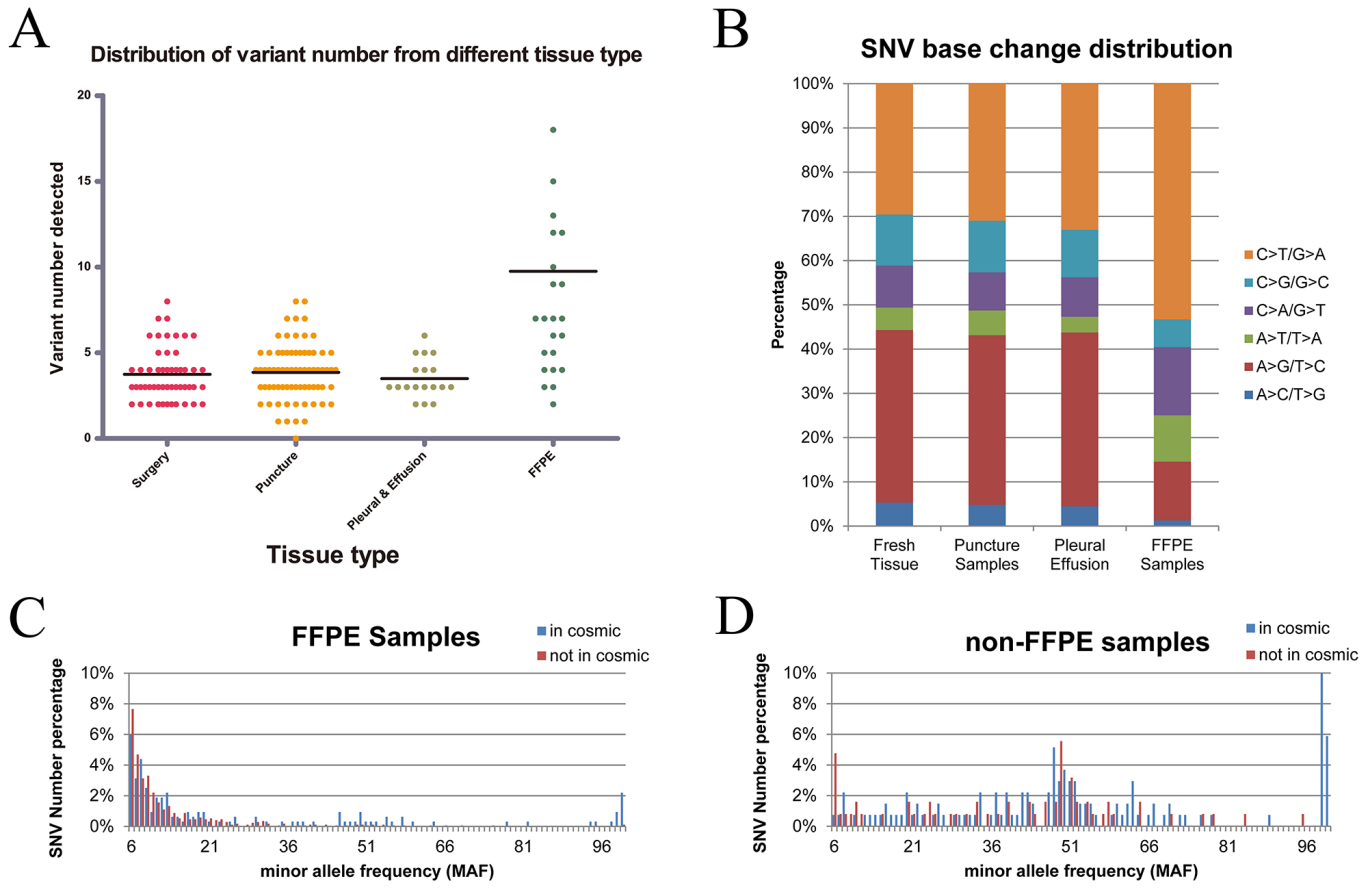
Distribution of variant numbers in different sample types and the distribution of variations in FFPE tumor samples **(A)** Distribution of variant numbers in different sample types. The black lines are the median number for the variant number of the corresponding sample types. The amounts of genetic variants per sample of FFPE samples were nearly twofold higher than those of the other fresh samples. **(B)** FFPE tumor samples included more C > T/G > A and fewer A > G/T >C mutations. **(C)** Most of SNVs. detected in the FFPE tumor samples were concentrated at the lowest MAF (less than 0.1) and were not included in the COSMIC database. **(D)** MAF of SNVs detected in the non-FFPE tumor samples demonstrates a similarly bi-modal distribution owing to less mutations with low allele frequency.

In this study, four types of tumor samples were collected, including surgically resected tumor tissues, puncture biopsy tumor tissues, tumor pleural effusion, and formalin-fixed paraffin-embedded (FFPE) tumor tissues. Different sample types displayed distinct mutation profiling. No statistically significant difference exists on the amounts of mutations per sample among the three fresh tumor tissue samples, namely, surgically resected tumor tissues (median, 3; range, 2–8), puncture biopsy tumor tissues (median, 4; range, 0–8), and tumor pleural effusion (median, 3; range 2–6). However, the number of genetic variants per FFPE tissue sample (median, 7; range, 2–18) was nearly twofold higher than the others by nonparametric test (*p* = 0.006, Figure 3A). Different from the mutations in fresh tumor samples, many variants detected in FFPE tumor tissues were at relatively lower coverage and minor allele frequency (MAF), including more C > T/G > A mutations (Figure 3B), and were not included in the Catalogue of Somatic Mutations in Cancer (COSMIC) database (Figure 3C, 3D). Most of non-COSMIC, low-frequency, and C > T mutations detected in FFPE tumor tissues were not verified as real using polymerase chain reaction (PCR) assay or Sanger sequencing method. Therefore, disparity in DNA integrity between fresh and formalin-fixed tumor tissues significantly affected tumor mutation profiling.

### Concurrent somatic mutations in multiple driver genes were frequently detected in solid tumor tissues

**Figure 4:**
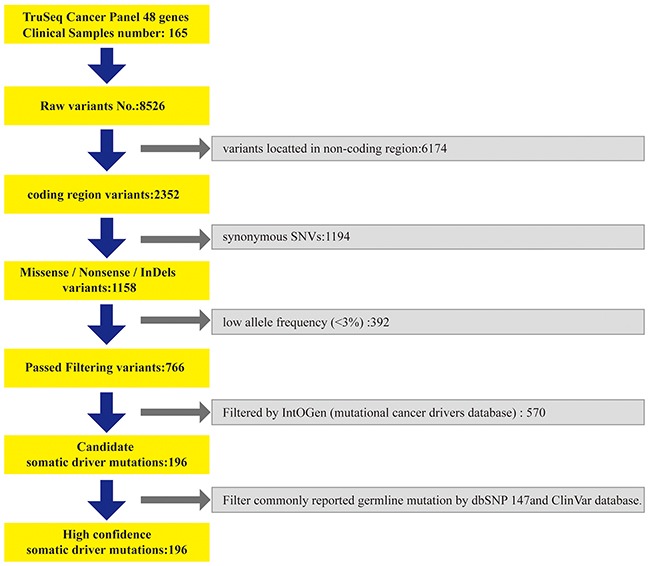
The data analysis processing

We further filtered the 766 variants by IntOGen cancer drivers database, and then data were filtered by the commonly reported germline mutation dbSNP (release 147) and ClinVar database. In the end, we obtained 196 somatic driver mutations for subsequent analysis (Figure 4).

**Figure 5:**
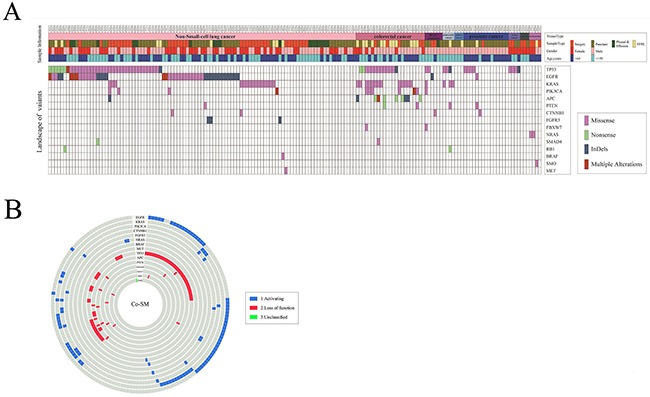
Distribution of 196 driver mutations **(A)** The top of the figure shows the tissue type, sample type, gender, and age for 165 samples. The middle of the figure shows 196 variants distributed based on the sample ID and gene name. **(B)** The figure shows the co-occurrence of driver mutations in samples.

Among the 196 somatic driver mutations in 165 samples, 15 driver genes were included, such as TP53, EGFR, KRAS, PIK3CA, APC, PTEN, CTNNB1, FGFR3, FBXW7, NRAS, SMAD4, RB1, BRAF, SMO, and MET (Figure 5A). These driver genes can be divided into two parts, as follows: activating mode of action driver (A-driver) genes and loss-of-function mode of action driver (LF-driver) genes. Those somatic mutations were mainly concentrated on certain A-driver genes, such as EGFR (27.88%, 46/165), KRAS (19.39%, 32/165), PIK3CA (6.67%, 11/165), and CTNNB1 (2.42%, 4/165), and on some LF-driver genes, such as TP53 (38.79%, 64/165), APC (6.06%, 10/165), and PTEN (3.03%, 5/165) (Supplementary Table 2). Of these samples, 74.5% (123/165) displayed at least one type of somatic mutation in one gene, either missense SNV, nonsense SNV, or InDels. Given that some tumor types had fewer samples for the lower incidence, the concurrent somatic driver mutations were focused on the top two tumors NSCLC and CRC.

As shown in Figure 5A, 77.7% (80/103) of NSCLC samples carried somatic mutations on driver genes, and a total of 120 driver gene mutations were distributed among various driver genes with different modes of action. Additionally, most of the somatic mutations were distributed among A-driver genes. Nearly 65% of NSCLC samples possessed somatic mutations on A-driver genes, which were mainly focused on EGFR (42.72%, 38/103), KRAS (14.56%, 15/103), PIK3CA (3.88%, 4/103), and FGFR3 (2.91%, 3/103). Somatic mutations on LF-driver genes were also detected, which were mainly concentrated on TP53 (36.89%, 38/103). Similarly, in CRC, 95.65% (22/23) of the samples were carrying 47 somatic mutations on multiple driver genes (Figure 5A), which mainly focused on the A-driver genes, KRAS (56.52%, 13/23) and PIK3CA (26.09%, 6/23), and on the LF-driver genes, TP53 (56.52%, 13/23), APC (39.13%, 9/23), and PTEN (8.7%, 2/23).

However, 74.5% (123/165) of the solid tumors had more than one somatic mutation in either one or multiple genes. The concurrent somatic mutations (Co-SM) in multiple driver genes were frequently detected in solid tumor tissues, among which 39% (48/123) of the samples were bearing Co-SM in multiple driver genes. As shown in Figure 5B, 28.16% (29/103) of NSCLC samples have Co-SM, among which 79.31% (23/29) were distributed on both A-driver genes and LF-driver genes. By contrast, only 17.24% (5/29) of NSCLC samples had Co-SM exclusively in A-driver genes, whereas 3.45% (1/29) of NSCLC samples had Co-SM exclusively in LF-driver genes. Therefore, Co-SM of A-driver genes and LF-driver genes were the most common type. Similarly, in CRC, 65.22% (15/23) of samples had more than one driver gene mutation, and 79.31% (12/15) possessed Co-SM in both A-driver and LF-driver genes. By contrast, only 13.33% (2/15) of CRC samples had Co-SM exclusively in A-driver genes, whereas 6.67% (1/15) of CRC samples had Co-SM exclusively in LF-driver genes (Supplementary Table 3). Moreover, we compared the distribution of Co-SM in three main tumor types, namely, NSCLC, CRC, and PsC, which were significantly different by nonparametric test (*p* = 0.000). The combinations of EGFR + TP53 (37.5%, 18/48), KRAS + TP53 (10.4%, 5/48), and KRAS + APC (8.3%, 4/48) were the most frequently detected Co-SM in solid tumor tissues. Furthermore, 8% (10/123) of the samples had Co-SM solely in one driver gene, such as EGFR.

### Co-SM in driver genes were correlated with lymph node metastasis and pathological types in NSCLC

The association between Co-SM in driver genes and clinical–pathological features was analyzed in NSCLC. A total of 103 cases of patients with NSCLC were followed for 2 years, among which 12 patients were lost during follow-up. The clinical and pathological features of 91 patients with NSCLC were compared and analyzed (Table 1). Based on the NGS results, 91 patients with NSCLC were divided into two groups: mutation-positive (M+, 71 cases) patients and mutation-negative (M−, 20 patients) patients. No significant difference was identified among gender, age, tumor location, clinical stage, pathology typing, and lymph node and/or distant metastasis status between M+ and M− patients. We further divided the 71 patients into two subgroups: patients harboring Co-SM (Co-SM+, 45 patients) and patients harboring single mutation (Co-SM−, 26 patients). We found that a higher frequency of lymph node metastasis occurred in Co-SM+ patients compared with Co-SM− patients, with marginal significance (*p* = 0.119, chi-squared test). Particularly, 43% of Co-SM+ patients had lymph node metastasis in contrast to 23% of Co-SM− patients. Co-SM+ patients displayed different pathological features compared with Co-SM− patients. More adenocarcinoma rather than squamous carcinoma occurred in Co-SM+ patients than in Co-SM− patients (45.45% vs. 6.25%) with p value of 0.014 by Fisher's exact test. Furthermore, Co-SM in EGFR gene, especially both sensitive and resistant mutations, such as L858R + T790M or Exon19 deletion + T790M, was common in Co-SM+ adenocarcinoma. The distribution was significantly different between untreated naive patients with NSCLC and patients with refractory cancer after multiple treatments, including chemotherapy and target therapy by Fisher's exact test (0% vs. 26.32%, *p* = 0.02).

**Table 1:** Association of driver gene mutations and clinical-pathological characteristics of NSCLC

Characteristics	No. of patients (N =71)		P-value
	Mutation+	Mutation−	
**Sex**			
Male	12	22	P=1
Female	14	23	
**Age**			
>60	11	23	P=0.623
≤60	15	22	
**Tumor location**			
Left lung	14	15	P=0.133
Right lung	12	30	
**Stage**			
I	4	13	P=0.538
II	3	4	
III	5	5	
IV	14	23	
**Lymph node metastasis**			
Yes	21	28	P=0.119
No	5	17	
**Distant metastasis**			
Yes	12	18	P=0.628
No	14	27	
**Pathology**			
Adenocarcinoma	25	30	P=0.014
Squamouscarcinoma	1	11	
Large cell	0	4	
**Treatment**			
Niave	17	25	P=0.462
Refractory	9	20	

The correlation between Co-SM and lymph node status was validated with TCGA database. We studied data from 478 patients with lung adenocarcinoma from the publication by TCGA database. To ensure that the TCGA data were consistent with our results, the TCGA mutation data were focused on the 48 genes listed in our study and filtered using IntOGen, dbSNP (release 147), and ClinVar databases accordingly to obtain the final list of somatic driver mutations. Finally, 367 patients were kept with a total of 637 mutations with at least one mutation per person. Even though patients in the TCGA database are mostly Caucasians rather than Mongolians, a higher frequency of Co-SM+ occurred in patients with lymph node metastasis in comparison with patients without lymph node metastasis (*p* = 0.012 by chi-squared test). In particular, 39% (50/127) of patients with lymph node metastasis had Co-SM+ in comparison with 26% (63/240) of patients without lymph node metastasis. The TCGA data are further supportive evidences to strengthen our observation.

## DISCUSSION

Before using TSACP, we have assessed the specificity and sensitivity of TSACP in our previous study. The lowest threshold of genetic mutation detection (both SNVs and InDels) is 3% at the depth of 2000× for TSACP. Furthermore, several conventional genetic analysis methods, including quantitative polymerase chain reaction, Sanger sequencing, and pyrosequencing, were compared with NGS assay to validate the detected variants in this cohort. A total of 20 variants were validated, and these variants were completely consistent among NGS and other genetic analysis methods (Supplementary Table 4). In our study, we verified the final 196 variants for subsequent clinical–pathological analysis using PCR method. The IntOGen cancer drivers, dbSNP, and ClinVar databases were used to exclude commonly reported germline mutations (such as the recurrent mutations in KDR gene) and FFPE false-positive mutations. We also adopted VarScan2 mutation calling pipeline to avoid false-positive mutations.

The patterns of mutations in well-studied oncogenes and tumor suppressor genes are highly characteristic and nonrandom. Vogelstein et al. supplied a lenient “20/20 rule” to classify oncogenes and tumor suppressor genes and published a list of 125 gene (Vogelstein, Papadopoulos et al. 2013). In our manuscript, we classified 196 somatic driver mutations into two parts: A-driver genes and LF-driver genes using IntOGen cancer driver database. From the perspective of oncogenes and tumor suppressor genes, we reanalyzed our results based on the frequently-used tumor-associated gene database. We compared our results with Vogelstein's results; all the genes had consistent classification except gene SMO, which was classified as an oncogene in Vogelstein's paper but a tumor suppressor gene in the tumor-associated gene database we used.

Chang et al. developed a statistical algorithm to identify recurrently mutated residues in tumor samples. They applied the algorithm to 11,119 human tumors, spanning 41 cancer types, and identified 470 somatic substitution hotspots in 275 genes, including 243 novel hotspots [16]. We compared our data with the published 470 somatic substitution hotspots, and 14 mutations were previously filtered out by the IntOGen database. The 14 mutations may confer a selective growth advantage to the tumor cell but need further confirmation.

We further studied the mutation abundance of TSACP and whole exome sequencing (WES) using the public data of 478 NSCLC and 472 CRC cases in TCGA database. We firstly compared the mutation abundance of 48 genes covered by TSACP between our dataset and TCGA database, and found that no significant differences were detected in normalized numbers of mutations per 100kb either in NSCLC or CRC samples (*p*=0.966; *p*=0.346). Afterwards, we compared the mutation abundance of 48 selected genes and total genes in TCGA database, and found that in NSCLC the normalized numbers of mutations of 48 genes (medican:3.7 vs 0.6 per 100kb, *p* =0.000) were statistically higher than those of WES using nonparametric test. Similar result was obtained in CRC (medican:7.4 vs.0.38 per 100kb, *p*=0.000). TSACP is to detect somatic mutations within important cancer-related hotspot genes, covering quite a number of highly mutated loci in cancer. Comparing to WES, higher mutation abundance was detected using TSACP, which implied that TSACP is relatively more efficient than WES in screening driver gene mutations and predicting clinical response to target therapy and immunotherapy.

In our study, 74.5% of the solid tumors bear more than one somatic mutation in either one or multiple genes, which showed significant medical value. Previous studies have demonstrated that the Co-SM in multiple driver genes were correlated with certain clinical–pathological features of solid tumors and therapeutic response to certain treatments; for example, KRAS and PIK3CA comutations were the most distinctive features of early NSCLC, whereas the RB1 and TP53 coalterations indicated a characteristic genotype of small cell lung cancer [17]. In breast cancer, TP53 + PIK3CA comutations conferred the worst disease-free survival in patients than single PIK3CA mutation [18]. In NSCLC, single PIK3CA mutation-bearing patients had shorter overall survival than those with the PIK3CA + EGFR or PIK3CA + KRAS comutations [19]. In our study, Co-SM in multiple driver genes was correlated with lymph node metastasis and pathological typing of NSCLC. Higher frequency of lymph node metastasis and higher percentage of adenocarcinoma occurred in Co-SM+ patients with NSCLC in comparison with Co-SM− patients. Medical significance of Co-SM with lymph node status in NSCLC was validated in TCGA database. The TCGA database mainly included white people, which was different from our collection that consisted of Asian people. TCGA data showed that Co-SM+ occurred more in patients with lymph node metastasis than in patients without lymph node metastasis, this finding further strengthened our results. Hence, NSCLC bearing Co-SM in multiple driver genes might be more aggressive and lead to a higher risk of early relapse after surgery, for which more comprehensive treatment should be prescribed to control disease progression and improve prognosis. Our research showed wider significance, which is not limited to Asian populations.

In our study, concurrent sensitive and resistant somatic mutations in EGFR gene were the most frequently detected Co-SMs in lung adenocarcinoma, in which L858R + T790M or Exon19 deletion + T790M were frequently detected in patients with refractory NSCLC rather than in untreated naive patients; this result was consistent with the knowledge that most of the secondary EGFR-TKIs resistance are correlated with emerging EGFR T790M-positive tumor clones in NSCLC. Given the deep coverage of the platform, we did the abundance-based clonality analysis for the EGFR L858R+T790M to distinguish which one was clone or subclone according to the reported method [20, 21]. Clonality analysis was performed to distinguish which one was cloned or subcloned. Subclones were obtained by clustering cancer cell fractions by PyClone, which can deconvolute the tumor into subclones using a hierarchical Bayesian clustering mode. The subclones were compared using the density plot of cancer cell fractions. EGFR L858R+T790M mutation was detected in two patients with NSCLC in our study, in which L858R was demonstrated as the major subclone and T790M as the minor subclone. The above results were consistent with previous report that T790M mutation was detected as a “second-site mutation” in EGFR-mutated lung cancers, which is prone to acquire resistance to erlotinib or gefitinib [22, 23].

Moreover, in our study, we further studied the influence of formalin-fixed conditioning of tumor tissues on NGS data. Weyn et al. reported the significant association between the storage of the FFPE blocks and the result quality that lower DNA quality and a high number of false mutations were observed in tumor tissue samples older than 4 years [20]. It was accepted that the storage period of the FFPE blocks significantly influenced the integrity of DNA samples and efficiency of PCR amplification in NGS [21]. Furthermore, Ivanov et al. reported that lower concentration of pre-normalization libraries in FFPE samples dramatically increased the number of sequence artifacts after NGS [22]. Accordingly, we also found more variants in formalin-fixed tumor tissues, and most of them were C > T mutations with low frequency, which was significantly consistent with a previous report stating that more potential artifacts occurred in FFPE tumor tissues than in fresh tissues [19]. To eliminate the contamination of false mutations, we conducted gene screening filtered by IntOGen cancer drivers database, dbSNP 147, and ClinVar database, which excluded commonly reported germline mutations and FFPE artifacts before the subsequently clinical–pathological analysis. However, we did not find significant correlation between archival age of FFPE samples and mutation numbers in our study (*p* = 0.813). This might be derived from short storage time of in-house FFPE samples, which are less than 4 years.

We further studied the correlation between the proportions of C:G>T:A mutations and archival age of FFPE samples. FFPE samples in our study were divided into two groups using the median archival age of FFPE samples (30.5days) as cutoff. There was no significant difference in the proportions of C:G>T:A mutations between two groups. But when we conducted the Spearman correlation analysis on the archival age of FFPE samples and the proportions of C:G>T:A mutations, we found a trend of increased proportions of C:G>T:A mutations in FFPE samples of longer archival age with a marginal significance (p = 0.145). Therefore, the mutation signature analysis might help to evaluate the negative influence of archival age of FFPE samples on NGS since the FFPE-associated false positives have specific sequence contexts.

TSACP-based target NGS can provide high efficiency in detecting low-frequency drug-sensitive genetic mutations in advanced patients who might be beneficial from the target therapies. The typical case was listed below. A 53-year-old male IV-stage NSCLC patient pleural fluid was sequenced by NGS; EGFR 19 exon deletions and EGFR T790M were both detected, and their variant frequencies were 15.66% and 4.58% covered, respectively, at around 2000× sequencing depth. The patient was treated with Afatinib for 1 month, and the tumors shrunk at least 10%. The clinical symptoms were relieved accordingly. In this case, the EGFR T790M mutation was of low frequency, which can be only detected at the sequencing depth of 2000×, in comparison with whole genome sequencing (Supplementary Figure 1).

In conclusion, our study demonstrated that tumor genetic profiling varied significantly among different tumor and sample types. However, Co-SM in multiple driver genes with different modes of action were frequently detected in solid tumor tissues and correlated with lymph node metastasis, pathological typing, and clinical response in NSCLC. The coordinating network among driver genes is playing more vital roles in carcinogenesis than individual driver genes. However, enlarging the sample size of this study will provide more evidences to elucidate further the interaction among multiple genes during carcinogenesis and progression.

## MATERIALS AND METHODS

### Clinical samples

A total of 165 solid malignancy samples, including 103 NSCLC, 23 CRC, 15 PsC, 6 HC, 4 ovarian cancer (OC), 4 melanoma (MA), 4 pancreatic cancer (PcC), 3 GC, and 3 sarcoma, were collected from the Department of Pathology of Tianjin Medical University Cancer Institute & Hospital (TMUCH) between August 2013 to January 2016. Among them, 51 were surgically resected tumor samples, 72 were puncture biopsy tumor samples, 18 were pleural effusion samples, and 24 were FFPE tumor samples. All samples had a minimum tumor content of 70%, and the detectable levels of tumor cells in pleural fluid were independently assessed by two experienced pathologists at TMUCH. This project was approved by the Ethics Committee of Tianjin Medical University. All experiments were performed in accordance with the principles of the Declaration of Helsinki. Written consents were obtained from each patient.

### Library construction and amplicon-based target NGS

For fresh tumor tissues, such as surgically resected tumor samples, puncture biopsy tumor samples, and pleural effusion samples, genomic DNA was extracted using the QIAamp DNA Mini Kit (Qiagen, Hilden, Germany). For FFPE tissues, genomic DNA was extracted using QIAamp FFPE DNA Tissue Kit (Qiagen, Hilden, Germany). Sequencing libraries were generated using TSACP (Illumina, San Diego, CA, USA), a previously validated targeted gene panel covers mutational hotspots in 48 cancer-related genes based on amplicons sequencing, which consists of 212 pairs of probes designed to bind genomic target sequences of interest spanning more than 35 kb length of genomic DNA [24–25]. Sequencing was performed on a MiSeq sequencer with paired-end reads (MiSeq Reagent Nano Kit v2, 300 cycles). The designed average sequencing depth was ×2000.

### Variant calling and bioinformatics analyses

The generation of FASTQ files from raw read data was accomplished with MiSeq Reporter Software 2.6 (Illumina). The alignment of paired-end raw reads to the human hg19 genome assembly was performed using BWA aligner 0.7.15. The Somatic Variant Caller Algorithm Version 3.1.10.0 (Illumina) was used for variant identification from aligned reads, and the coverage analysis was performed in parallel with Samtools 1.3.1. Variants were annotated by ANNOVAR (2016Feb01) with dbSNP release 147. Nonprotein-coding region variants and synonymous variants were excluded in this study. Variants with allele frequency lower than 3% were filtered out, which were considered low-quality data based on our previous study. Somatic driver mutations were identified using several databases, which are IntOGen, dbSNP release 147, and ClinVar databases. A-driver genes and LF-driver genes were classified using IntOGen cancer driver database provided data The Integrative Genomics Viewer 16 (Broad Institute, Cambridge, Massachusetts) was used to visualize variants against the reference genome. VarScan2 mutation calling pipeline was used for validation to avoid false positives. TCGA data were obtained from official website. TCGA mutations data among the 48 genes used in TSACP were picked and then filtered with IntOGen, dbSNP release 147, and ClinVar database the same way as TSACP data. Clonality analysis was performed using PyClone.

### Statistical analysis

The SPSS 19.0 software was used for statistical analyses. Descriptive statistics calculated the median values and ranges of the variant number. Nonparametric tests were used to compare the distribution of variant number in different tumor and sample types, in which Kruskal–Wallis test was followed by all pairwise multiple comparisons. Chi-squared or Fisher's exact test was used to compare variant numbers among groups with different clinical and pathological characteristics. All tests were two tailed, and *p* ≤ 0.05 was considered statistically significant.

## SUPPLEMENTARY MATERIALS FIGURES AND TABLES





## Abbreviations

NGS: next-generation sequencing
TSACP: TruSeq Amplicon Caner Panel
FFPE: formalin fixed paraffin embedded
NSCLC: non-small cell lung cancer
CRC: colorectal cancer
PsC: prostate cancer
HC: hepatic cancer
OC: ovarian cancer
MA: melanoma
PcC: pancreatic cancer
GC: gastric cancer
TMUCH: Tianjin Medical University Cancer Institute & Hospital
A-driver: activating mode of action driver
LF-driver: loss-of-function mode of action driver
PCR: polymerase chain reaction
EGFR–TKI: epidermal growth factor receptor–tyrosine kinase inhibitor
MAF: minor allele frequency
WES: whole exome sequencing
