# Supplementary material for: Concurrent somatic mutations in driver genes were significantly correlated with lymph node metastasis and pathological types in solid tumors
Source: Oncotarget. 2017 Aug 7;8(40):68746–57. doi: 10.18632/oncotarget.19975 (PMC5620293; doi:10.18632/oncotarget.19975)
Supplement: Supplementary file 1 [file oncotarget-08-68746-s001.pdf]

Concurrent somatic mutations in driver genes were significantly correlated with lymph node metastasis and pathological types in solid tumors

SUPPLEMENTARY MATERIALS

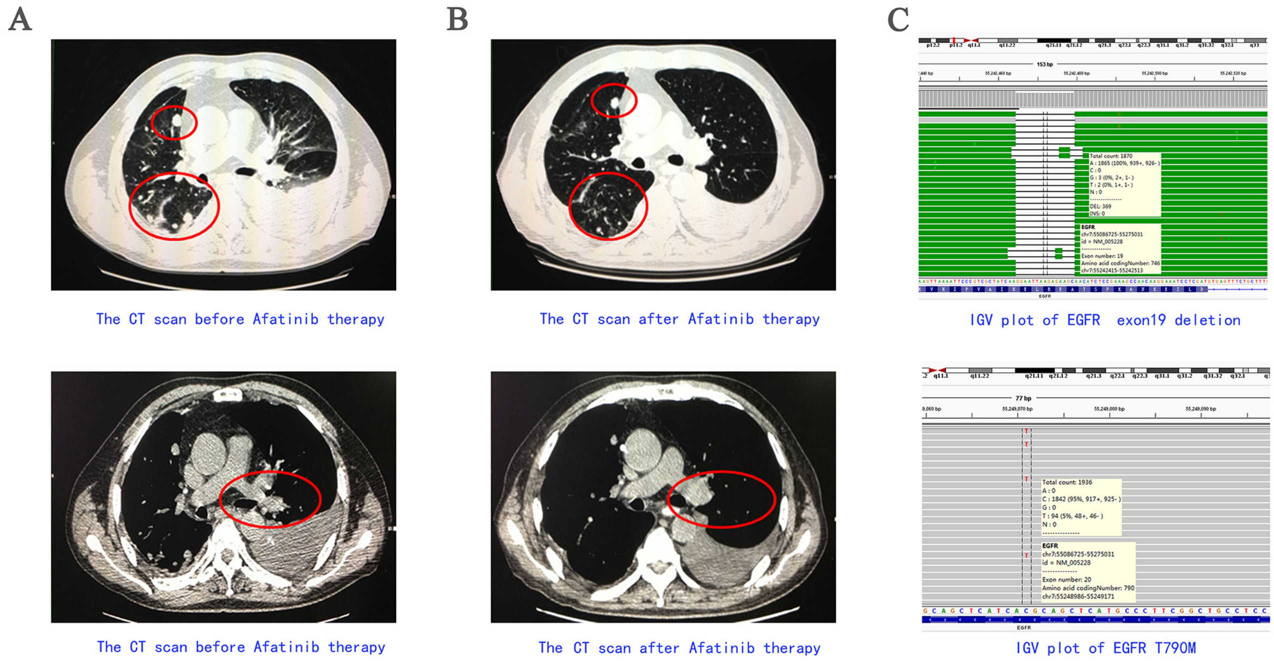

Supplementary Figure 1: (A) The CT scan before Afatinib therapy of the patient. **(B)** The CT scan after Afatinib therapy. **(C)** IGV plot shows the sequence overview of EGFR exon19 deletions and EGFR T790M in this case.

Supplementary Table 1: Mutations distribution in 48 genes among 9 cancer types.

See Supplementary File 1

Supplementary Table 2: Frequency of multiple driver gene mutations in solid tumor samples

Characteristics			Ratio of patients
Total			
	A-driver		
		EGFR	27.88%,46/165
		KRAS	19.39%,32/165
		PIK3CA	6.67%,11/165
		CTNNB1	2.42%,4/165
		FGFR3	1.82%,3/165
	LF-driver		
		TP53	38.79%,64/165
		APC	6.06%,10/165
		PTEN	3.03%,5/165
NSCLC			
	A-driver		
		EGFR	42.72%,38/103
		KRAS	14.56%,15/103
		PIK3CA	3.88%,4/103
		FGFR3	2.91%,3/103
	LF-driver		
		TP53	36.89%,38/103
CRC			
	A-driver		
		KRAS	56.52%,13/23
		PIK3CA	26.09%,6/23
	LF-driver		
		TP53	56.52%,13/23
		APC	39.13%,9/23
		PTEN	8.7%,2/23

Supplementary Table 3: Frequency of multiple Co-SM in solid tumor samples

Characteristics		Ratio of patients
somatic mutation +		74.5%(123/165)
Co-SM +		39%(48/123)
	EGFR+TP53	37.5%(18/48)
	KRAS+TP53	10.4%(5/48)
	KRAS+APC	8.3%(4/48)
	EGFR+EGFR	8% (10/123)
NSCLC		28.16%(29/103)
	A+LF	79.31%(23/29)
	A-only	17.24%(5/29)
	LF-only	3.45%(1/29)
CRC		65.22%(15/23)
	A+LF	79.31%(12/15)
	A-only	13.33%(2/15)
	LF-only	6.67%(1/15)

Supplementary Table 4: Validation of variants detected by NGS by conventional genetic analysis method

Sample	Gene	Chromosome	Position	ref	alt	Mutation by NGS(coverage, frequency)	Validation of mutation	Validation method
T00006	SMARCB1	22	24145675	G	C	Mut(958,48.64%)	Mut	Sanger Sequencing
T00006	PDGFRA	4	55152040	C	T	Mut(1755,49.06%)	Mut	Sanger Sequencing
T00009	ALK	2	29432776	T	C	Mut(2015,51.76%)	Mut	Sanger Sequencing
T00008	NRAS	1	115256529	T	C	Mut(249,52.61%)	Mut	Sanger Sequencing
T00022	APC	5	112175770	G	A	Mut(3003,65.23%)	Mut	Sanger Sequencing
T00022	TP53	17	7579472	G	C	Mut(499,73.95%)	Mut	Sanger Sequencing
T00019	GNA11	19	3114864	C	T	Mut(184,76.63%)	Mut	Sanger Sequencing
T00016	FGFR1	8	38282294	C	T	Mut(1265,83.48%)	Mut	Sanger Sequencing
T00010	APC	5	112175770	G	A	Mut(2375,99.49%)	Mut	Sanger Sequencing
T00026	PDGFRA	4	55141055	A	G	Mut(1209,99.67%)	Mut	Sanger Sequencing
T00012	APC	5	112175770	G	A	Mut(2834,24.45%)	Mut	qPCR
T00016	FLT3	13	28610183	A	G	Mut(209,25.84%)	Mut	qPCR
T00019	JAK3	19	17945729	A	G	Mut(1213,23.66%)	Mut	qPCR
T00020	KRAS	12	25398284	C	G	Mut(1667,20.76%)	Mut	qPCR
T00021	CTNNB1	3	41266101	C	G	Mut(1211,8.18%)	Mut	qPCR
T00015	CDKN2A	9	21971261	A	C	Mut(24,17%)	Mut(6%)	Pyrosequencing
T00024	PIK3CA	3	178936091	G	A	Mut(1816,15%)	Mut(24%)	Pyrosequencing
T00010	KIT	4	55593464	A	C	Mut(2570,22%)	Mut(16%)	Pyrosequencing
T00028	PIK3CA	3	178952085	A	G	Mut(1771,6%)	Mut(6%)	Pyrosequencing
T00010	SMARCB1	22	24176408	G	C	Mut(581,29%)	Mut(2%)	Pyrosequencing
