# Supplementary material for: Concurrent somatic mutations in driver genes were significantly correlated with lymph node metastasis and pathological types in solid tumors
Source: Oncotarget. 2017 Aug 7;8(40):68746–57. doi: 10.18632/oncotarget.19975 (PMC5620293; doi:10.18632/oncotarget.19975)
Supplement: Supplementary file 2 [file oncotarget-08-68746-s002.docx]

| Supplementary Table 1: Mutations distribution in 48 genes among 9 cancer types | | | | | | |  |  |  |
| --- | --- | --- | --- | --- | --- | --- | --- | --- | --- |
| Characteristics | 9 cancer types | | | | | | | | |
|  | NSCLC | Colorectal cancer | Prostate cancer | Hepatic cancer | Ovarian cancer | Pancreatic cancer | Melanoma | Gastric cancer | Sarcoma |
| TP53 | 87.4% | 95.7% | 93.3% | 50.0% | 100.0% | 100.0% | 100.0% | 100.0% | 66.7% |
| KDR | 78.6% | 56.5% | 53.3% | 66.7% | 50.0% | 100.0% | 75.0% | 100.0% | 66.7% |
| EGFR | 44.7% | 8.7% | 6.7% | 16.7% | 0.0% | 0.0% | 0.0% | 0.0% | 0.0% |
| KRAS | 15.5% | 56.5% | 0.0% | 16.7% | 0.0% | 75.0% | 0.0% | 0.0% | 0.0% |
| MET | 16.5% | 17.4% | 33.3% | 16.7% | 50.0% | 25.0% | 0.0% | 33.3% | 0.0% |
| STK11 | 17.5% | 13.0% | 0.0% | 16.7% | 0.0% | 25.0% | 0.0% | 0.0% | 0.0% |
| APC | 1.9% | 60.9% | 13.3% | 16.7% | 0.0% | 0.0% | 0.0% | 0.0% | 0.0% |
| FGFR3 | 9.7% | 17.4% | 6.7% | 0.0% | 25.0% | 0.0% | 0.0% | 0.0% | 0.0% |
| PIK3CA | 3.9% | 30.4% | 6.7% | 33.3% | 0.0% | 25.0% | 25.0% | 0.0% | 0.0% |
| KIT | 8.7% | 8.7% | 6.7% | 0.0% | 0.0% | 0.0% | 25.0% | 33.3% | 33.3% |
| GNA11 | 10.7% | 13.0% | 0.0% | 0.0% | 0.0% | 0.0% | 25.0% | 0.0% | 0.0% |
| ATM | 2.9% | 17.4% | 13.3% | 0.0% | 0.0% | 25.0% | 25.0% | 33.3% | 0.0% |
| MLH1 | 7.8% | 4.3% | 6.7% | 0.0% | 0.0% | 0.0% | 25.0% | 0.0% | 0.0% |
| PTEN | 4.9% | 13.0% | 6.7% | 0.0% | 0.0% | 25.0% | 0.0% | 33.3% | 0.0% |
| FBXW7 | 3.9% | 13.0% | 0.0% | 16.7% | 0.0% | 25.0% | 25.0% | 0.0% | 0.0% |
| HNF1A | 3.9% | 13.0% | 6.7% | 0.0% | 0.0% | 25.0% | 0.0% | 33.3% | 0.0% |
| ABL1 | 3.9% | 8.7% | 0.0% | 16.7% | 0.0% | 25.0% | 25.0% | 0.0% | 0.0% |
| SMAD4 | 2.9% | 21.7% | 0.0% | 0.0% | 0.0% | 0.0% | 0.0% | 0.0% | 0.0% |
| HRAS | 2.9% | 4.3% | 13.3% | 0.0% | 0.0% | 0.0% | 25.0% | 0.0% | 0.0% |
| RB1 | 2.9% | 4.3% | 0.0% | 0.0% | 0.0% | 25.0% | 25.0% | 33.3% | 0.0% |
| GNAQ | 1.9% | 4.3% | 0.0% | 0.0% | 0.0% | 25.0% | 25.0% | 33.3% | 0.0% |
| ERBB4 | 1.9% | 8.7% | 0.0% | 0.0% | 0.0% | 0.0% | 25.0% | 0.0% | 0.0% |
| NOTCH1 | 3.9% | 4.3% | 0.0% | 0.0% | 0.0% | 0.0% | 0.0% | 0.0% | 0.0% |
| NRAS | 1.0% | 4.3% | 0.0% | 0.0% | 0.0% | 0.0% | 75.0% | 0.0% | 0.0% |
| SMO | 1.9% | 8.7% | 0.0% | 0.0% | 0.0% | 0.0% | 25.0% | 0.0% | 0.0% |
| VHL | 1.0% | 13.0% | 0.0% | 0.0% | 0.0% | 25.0% | 0.0% | 0.0% | 0.0% |
| PDGFRA | 1.9% | 8.7% | 0.0% | 0.0% | 0.0% | 0.0% | 0.0% | 0.0% | 0.0% |
| RET | 1.9% | 4.3% | 0.0% | 0.0% | 0.0% | 25.0% | 0.0% | 0.0% | 0.0% |
| CTNNB1 | 1.9% | 0.0% | 6.7% | 16.7% | 0.0% | 0.0% | 0.0% | 0.0% | 0.0% |
| JAK3 | 1.0% | 0.0% | 6.7% | 0.0% | 0.0% | 0.0% | 25.0% | 0.0% | 0.0% |
| FGFR2 | 1.0% | 0.0% | 6.7% | 0.0% | 0.0% | 0.0% | 0.0% | 0.0% | 0.0% |
| FLT3 | 1.0% | 0.0% | 6.7% | 0.0% | 0.0% | 0.0% | 0.0% | 0.0% | 0.0% |
| BRAF | 1.0% | 0.0% | 0.0% | 0.0% | 0.0% | 0.0% | 0.0% | 0.0% | 0.0% |
| CDH1 | 0.0% | 0.0% | 0.0% | 0.0% | 25.0% | 0.0% | 0.0% | 0.0% | 0.0% |
| ERBB2 | 1.0% | 0.0% | 0.0% | 0.0% | 0.0% | 0.0% | 0.0% | 0.0% | 0.0% |
| JAK2 | 1.0% | 0.0% | 0.0% | 0.0% | 0.0% | 0.0% | 0.0% | 0.0% | 0.0% |
| SMARCB1 | 0.0% | 4.3% | 0.0% | 0.0% | 0.0% | 0.0% | 0.0% | 0.0% | 0.0% |
| CSF1R | 0.0% | 0.0% | 0.0% | 0.0% | 0.0% | 25.0% | 0.0% | 0.0% | 0.0% |
| PTPN11 | 0.0% | 0.0% | 0.0% | 0.0% | 25.0% | 0.0% | 0.0% | 0.0% | 0.0% |
| AKT1 | 0.0% | 0.0% | 0.0% | 0.0% | 0.0% | 0.0% | 0.0% | 0.0% | 0.0% |
| ALK | 0.0% | 0.0% | 0.0% | 0.0% | 0.0% | 0.0% | 0.0% | 0.0% | 0.0% |
| CDKN2A | 0.0% | 0.0% | 0.0% | 0.0% | 0.0% | 0.0% | 0.0% | 0.0% | 0.0% |
| FGFR1 | 0.0% | 0.0% | 0.0% | 0.0% | 0.0% | 0.0% | 0.0% | 0.0% | 0.0% |
| GNAS | 0.0% | 0.0% | 0.0% | 0.0% | 0.0% | 0.0% | 0.0% | 0.0% | 0.0% |
| IDH1 | 0.0% | 0.0% | 0.0% | 0.0% | 0.0% | 0.0% | 0.0% | 0.0% | 0.0% |
| MPL | 0.0% | 0.0% | 0.0% | 0.0% | 0.0% | 0.0% | 0.0% | 0.0% | 0.0% |
| NPM1 | 0.0% | 0.0% | 0.0% | 0.0% | 0.0% | 0.0% | 0.0% | 0.0% | 0.0% |
| SRC | 0.0% | 0.0% | 0.0% | 0.0% | 0.0% | 0.0% | 0.0% | 0.0% | 0.0% |
